# Generating genomic platforms to study *Candida albicans* pathogenesis

**DOI:** 10.1093/nar/gky747

**Published:** 2018-08-10

**Authors:** Mélanie Legrand, Sophie Bachellier-Bassi, Keunsook K Lee, Yogesh Chaudhari, Hélène Tournu, Laurence Arbogast, Hélène Boyer, Murielle Chauvel, Vitor Cabral, Corinne Maufrais, Audrey Nesseir, Irena Maslanka, Emmanuelle Permal, Tristan Rossignol, Louise A Walker, Ute Zeidler, Sadri Znaidi, Floris Schoeters, Charlotte Majgier, Renaud A Julien, Laurence Ma, Magali Tichit, Christiane Bouchier, Patrick Van Dijck, Carol A Munro, Christophe d’Enfert

**Affiliations:** 1 Fungal Biology and Pathogenicity Unit, Department of Mycology, Institut Pasteur, INRA, Paris 75015, France; 2 Medical Research Council Centre for Medical Mycology at the University of Aberdeen, Institute of Medical Sciences, Foresterhill, Aberdeen AB25 2ZD, UK; 3 VIB-KU Leuven Center for Microbiology, Leuven 3001, Belgium; 4 Laboratory of Molecular Cell Biology, KU Leuven, Leuven 3001, Belgium; 5 Univ. Paris Diderot, Sorbonne Paris Cité, Cellule Pasteur, Paris 75015, France; 6 Institut Pasteur-Bioinformatics and Biostatistics Hub-C3BI, USR 3756 IP CNRS-Paris 75015, France; 7 Modul-Bio, Parc Scientifique Luminy Biotech II, Marseille 13009, France; 8 Institut Pasteur-Biomics Pole-CITECH-Paris 75015, France

*Nucleic Acids Research*, gky594, https://doi.org/10.1093/nar/gky594

In the version of this article originally published online, an author's name was listed incorrectly. It was published as ‘Dijck, PV’ but should have been published as ‘Van Dijck, P.’ This has now been corrected online.

