# Supplementary material for: Generating genomic platforms to study Candida albicans pathogenesis
Source: Nucleic Acids Res. 2018 Jul 6;46(14):6935–49. doi: 10.1093/nar/gky594 (PMC6101633; doi:10.1093/nar/gky594)
Supplement: Supplementary Data [file gky594_supplemental_files.zip › ORFEOME_SuppTabS2.pdf]

Supplemental Table S2. Oligonucleotides used in this study

Name	Sequence (5' to 3')[1]
ARG4+	CCCCTTTAGTAAGATTTTTC
ARG4-	TAGTATTGGAGTACAAGGTA
207VER-F	TCGCGTTAACGCTAGCATGGATCTC
207VER-R	GTAACATCAGAGATTTTGAGACACG
SP3	CCCCccgcggACACTCTTTCCCTACACGACGCTCTTCCGATCTcctaggCCCCCgctagcAGCGCGGCTATCGTGGCT
SP4	GGGGccgcggGTGACTGGAGTTCAGACGTGTGCTCTTCCGATCctgatgctcttcgtccaga
oligo1_PciHABsrG	catgtATGGGTtacccatacgatgttcctgactatgcgggctatccctatgacgtcccggactatgcaggatcctatccatatgacgt tccagattacgctGCGATATCAACAAGTTT
oligo2_PciHABsrG	GTACAAACTTGTTGATATCGCagcgtaatctggaacgtcatatggataggatcctgcatagtccgggacgtcatagggatagcccgca tagtcaggaacatcgtatgggtaACCCATa
GTW02	TACGTTAACTCCTACACACATACAAATATAAATAATTTACAatgggttacccatacgatg
GTW03	GTCTTTCATTGCCATACGGA
GTW04	tttttacatgtATGGCTTCTAAAGGAGAAGAAC
GTW05	TTTTTTTTTGATATCTCACCACCTGGTCCACCTTTGTATAGTTCATCCATGCCATG
GTW07	TTAGTACTCCTACACACATACAAATATAAATAATTTACAatggcttctaaaggagaagaac
GTW13	tttttACATGTGATATCAatgaagagaagatggaaaaagaatttc
GTW14	ttttttgtacaaacttgttgaGGTTGACTTCCCCGCGGAA
GTW20	CGTTTCTCGTTCAGCTTTCTTGTACAAAGTGGtacccatacgatgttcctgac
GTW21	CTCTAATTTGTGAGTTTAGTATACATGCATTTAtataccatccttatctagagcgg
GTW11	GTGGTTGATATCAcgtacgctgcagggtgctggcg
GTW12	AAATCATTACGACCGAGATTCCCG
GTW15	tttcttgtacaaagtggttATATCAGAAAAGAGAAGATGG
GTW16	AGTATACAtgcaTTTACTTATAATGGC
SZ11	AATCCGCTCGAGCACACACATACAGACAATTGCG
SZ12	CCGGATATCGATTGTAAAGTTTGTTGATGTTAATTG
UZ50	gcgcccGACGTCggcgcgccagatctgttt
UZ51	gggccgGACGTCcgatgaattcgagctcgttt
CIpUL	ATACTACTGAAAATTTCCTGACTTTC
CIpUR	ATTACTATTTACAATCAAAGGTGGTC
CgSAT1-rev	GTGCGGCCATCAAAATGTAT
MTLa-5'F	GGATTCGAGCTCGCTCCTTTTAAATTAACC
MTLa-5'R	TAGGTGCGGCCGCATTATTGGTATTTGTCTTAA
MTLa-3'F	CCTAGCTCGAGTAACTCCACTGGAAATAAG
MTLa-3'R	GCCTAGGTACCGCAACTTAGATCAACAATAAAAGTC
MTLα-5'F	TACCAGAGCTCTAATAGTAGCATTCAGAT
MTLα-5'R	ATCATGCGGCCGCCTAACACAAAGCAATTTGGC
MTLα-3'F	TAGATCTCGAGAACATTAAATATATATTTAACTAGATCC
MTLα-3'R	TACTAGGTACCTGGGCCATGTTGGTTGCTAGAATTTGTC
WOR1F	GATTAGTCGACATGTCTAATTCAAGTATAG
WOR1R	GATGAAGATCTCTAAGTACCGGTGTAATAC

[1]lowercase
