# Supplementary material for: Generating genomic platforms to study Candida albicans pathogenesis
Source: Nucleic Acids Res. 2018 Jul 6;46(14):6935–49. doi: 10.1093/nar/gky594 (PMC6101633; doi:10.1093/nar/gky594)
Supplement: Supplementary Data [file gky594_supplemental_files.zip › ORFEOME_SuppTabS3.pdf]

Supplemental Table S3. ORFs with Ns tracts

Assembly22_Name	Assembly21_Name	Position of the Ns tract	Length of the Ns tract (Nt)	Cloned sequence	Current CGD sequence
C1_07030C	orf19.6202	752-772	21	CTGGTAACTTGCAACACTCTG	no more N's
C1_07880C	orf19.5059	354-391	38	**GGATTATTTATATATTGAAAAAAATATGATAATTCGAC**	**still contains N's**
C1_12220W	orf19.5242	100-119	20	ORF not validated in the ORFeome project	no more N's
C2_01830W	orf19.1492	1656-1669	14	ORF not validated in the ORFeome project	no more N's
C2_04320W	orf19.1590	1512-1519	8	ORF not validated in the ORFeome project	no more N's
		1636-1659	24	ORF not validated in the ORFeome project	
C2_06280C	orf19.5491	837-856	20	CGATTTTAAATATCGAGATT	no more N's
C3_01580W	orf19.1685	1313-1325	13	ORF not validated in the ORFeome project	still contains N's
C3_05390C	orf19.6977	1627-1659	33	**TGTTTTATTCCATTATTTGTTATATTATTGAAAT**	**still contains N's**
C4_01260W	orf19.4662	1499-1521	23	ORF not validated in the ORFeome project	no more N's
C4_02670W	orf19.2733	1161-1179	19	ATTTGAAAAATTATAAATTA	no more N's
C4_05320W	orf19.1789.1	878-926	49	**ATACTACTAATCCTCATAATGCAATCCCAGTATATGAAATTGCTACAGT**	**still contains N's**
C6_00170C	orf19.6319	1281-1303	23	ORF not validated in the ORFeome project	no more N's
CR_07070C	orf19.1816	2430-2440	11	ORF not validated in the ORFeome project	no more N's
C4_05280W	orf19.2629	2427-2456	30	ORF not validated in the ORFeome project	still contains N's
C2_03180C	orf19.5776	4896-4904	9	ORF not validated in the ORFeome project	no more N's
		4972-4977	6	ORF not validated in the ORFeome project	
