# Supplementary material for: Generating genomic platforms to study Candida albicans pathogenesis
Source: Nucleic Acids Res. 2018 Jul 6;46(14):6935–49. doi: 10.1093/nar/gky594 (PMC6101633; doi:10.1093/nar/gky594)
Supplement: Supplementary Data [file gky594_supplemental_files.zip › Supplemental Table Legends.docx]

**Supplemental Data**

Supplemental Table S1: Summary of the collection provided in this study

Supplemental Table S2: Oligonucleotides used in this study

Supplemental Table S3: ORFs with Ns tracts

Supplemental Table S4: IUPAC-containing ORFs validated in the *C. albicans* ORFeome

Supplemental Table S5: ORFs with recombined haplotypes
